# Consumer-Led Screening for Atrial Fibrillation: A Report From the mAFA-II Trial Long-Term Extension Cohort

**DOI:** 10.1016/j.jacasi.2022.07.006

**Published:** 2022-11-01

**Authors:** Yutao Guo, Hui Zhang, Gregory Y.H. Lip, mAF-App II Trial investigators

**Affiliations:** a Medical School of Chinese PLA, Department of Pulmonary Vessel and Thrombotic Disease, Sixth Medical Center, Chinese PLA General Hospital, Beijing, China; b Liverpool Centre for Cardiovascular Sciences, University of Liverpool and Liverpool Heart & Chest Hospital, Liverpool, United Kingdom; c Aalborg Thrombosis Research Unit, Department of Clinical Medicine, Aalborg University, Aalborg, Denmark

**Keywords:** atrial fibrillation, obstructive sleep apnea syndrome, photoplethysmography, prevalence, smart devices, AF, atrial fibrillation, AHI, apnea hypopnea index, CAD, coronary artery disease, ECG, electrocardiogram, mHealth, mobile health, OSA, obstructive sleep apnea, PPG, photoplethysmography

## Abstract

**Background:**

There are limited data on mobile health detection of prevalent atrial fibrillation (AF) and its related risk factors over time.

**Objectives:**

This study aimed to report the trends on prevalent AF detection over time and risk factors, with a consumer-led photoplethysmography screening approach.

**Methods:**

3,499,461 subjects aged over 18 years, who use smart devices (Huawei Technologies Co.) were enrolled between October 26, 2018, and December 1, 2021.

**Results:**

Among 2,852,217 subjects for AF screening, 12,244 subjects (0.43%; 83.2% male, mean age 57 ± 15 years) detected AF episodes. When compared with 2018, the risk (adjusted HRs, 95% CI) for monitored prevalent AF increased significantly for subjects when monitoring started in 2020 (adjusted HR: 1.34; 95% CI: 1.27-1.40; *P* < .001) or in 2021 (adjusted HR: 1.67; 95% CI: 1.59-1.76; *P* < 0.001). Of the 961,931 subjects who screening for both AF and OSA, 18,032 (1.9%, 97.8% male, mean age 44 ±17 years) were identified as high risk for OSA, which resulted in a 1.5-fold increase (95% CI: 1.30-fold to 1.75-fold) in the prevalent AF. A total of 5,227 (53.3%, 5,227/9,797) subjects were effectively followed up, from which 4,903 (93.8%, 4,903/5,227) subjects were confirmed with the diagnosis of AF, by the mAFA Telecare Team health providers.

**Conclusions:**

Photoplethysmography-based smart devices can facilitate screening for AF with >93% confirmation of detected AF episodes even for the low-risk general population, highlighting the increased risk for detecting prevalent AF and the need for modification of OSA that increase AF susceptibility. (Mobile Health [mHealth] Technology for Improved Screening, Patient Involvement and Optimizing Integrated Care in Atrial Fibrillation [mAFA (mAF-App) II study]; ChiCTR-OOC-17014138)

Atrial fibrillation (AF) is the most common arrhythmia with an ever-increasing public health burden and increasing health care costs.^1^, ^2^, ^3^ However, AF is commonly asymptomatic and some patients may have paroxysmal episodes. Asymptomatic AF recurrences are common, and may progress to clinical AF and an increased risk of adverse outcomes, including stroke.^4^^,^^5^ These unrecognized and untreated AF episodes would still lead to adverse outcomes, given a similarly poor prognosis of symptomatic and asymptomatic AF.^5^

The proliferation of mobile health (mHealth) and smart devices permits much earlier detection for AF, especially subclinical AF, in general population.^6^^,^^7^ Single/multilead electrocardiograms (ECGs), photoplethysmography (PPG), and oscillometry devices can be employed into the wearables to detect AF, with a validated diagnostic ability comparable to standard 12-lead ECGs.^8^^,^^9^ Greater AF detection has been associated with more prolonged, frequent monitoring.^9^ Indeed, PPG-based wristband/wearables have demonstrated the capability of screening for AF, comparable to a one-off 12-lead ECG.^8^

Smartwear devices used for mHealth may facilitate detection of AF, but there are limited data on mHealth detection of prevalent AF over time. The mAFA II (Mobile Health [mHealth] Technology for Improved Screening, Patient Involvement and Optimizing Integrated Care in Atrial Fibrillation) program, including the pre-mAFA phase of AF screening, also called the Huawei Heart Study, using Huawei smart devices. This phase investigated the incidence of AF identified with PPG-based screening strategy among the general population.^6^ Those with identified AF would be considered for entry into the mAFA II trial to validate the integrated ABC (avoid stroke with anticoagulants, better symptom management, cardiovascular and other comorbidities risk management) care supported by mHealth technology in the management of AF.

The present ancillary analysis from the mAFA-II Trial Long-Term Extension Cohort aimed to describe trends on prevalent AF detection in the general population over time with consumer-led screening for AF.

## Methods

The design and principal findings from the Huawei Heart Study have been previously reported.^6^ AF screening was conducted using PPG-based Huawei smart devices (Huawei Technologies Co.) in the general population. The monitored suspected AF cases were further confirmed by health providers in the mAFA Telecare center and network hospitals, with clinical evaluation, ECG, or 24-hour Holter monitoring. This screening approach has been previously reported in prior reports from this program.^6^^,^^10^

**Figure 1:**
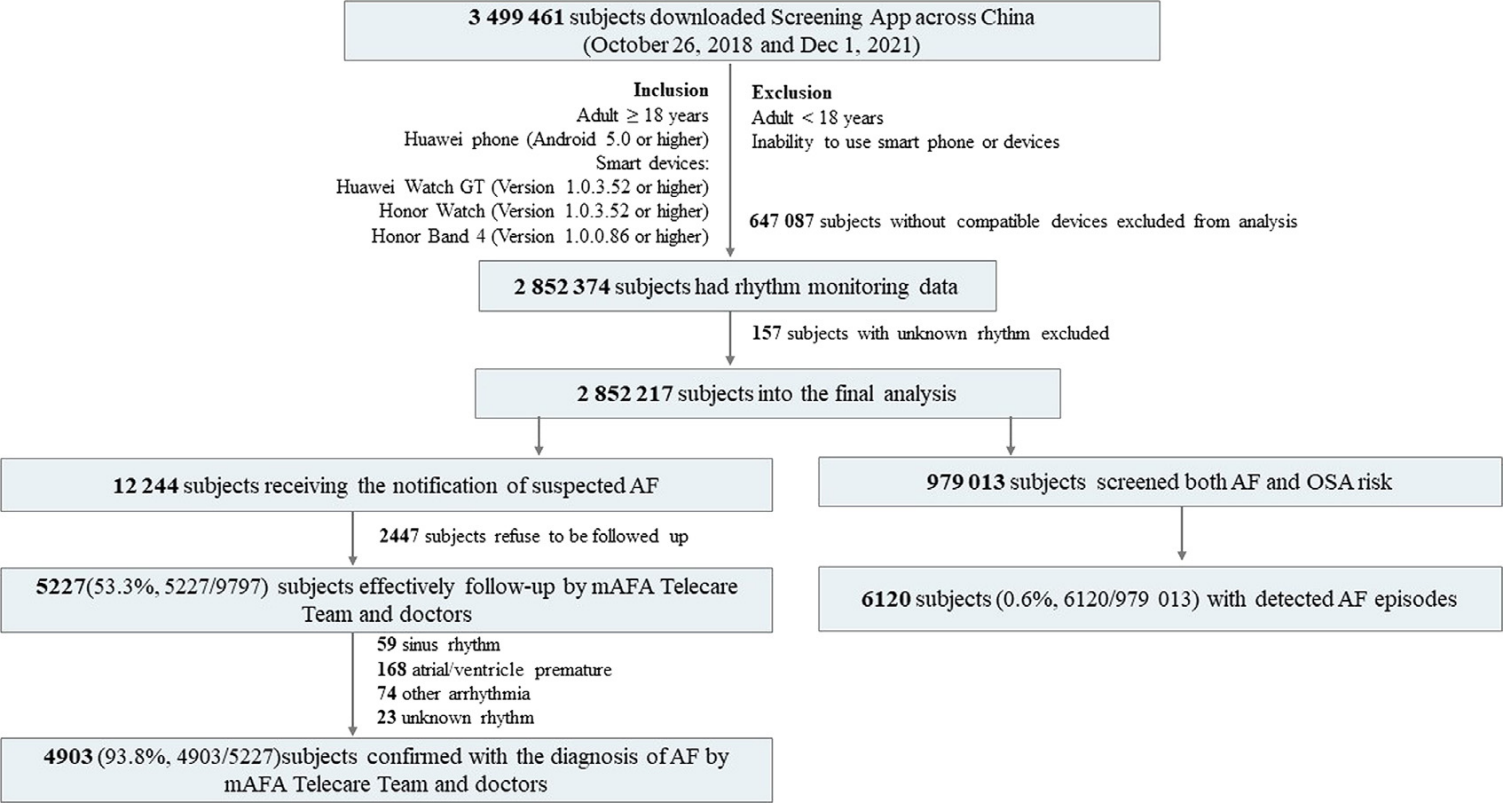
Flow Chart of Consumer-Led Screening for AF Between October 26, 2018, and December 1, 2021, there were 3,499,461 subjects who downloaded the mobile Atrial Fibrillation Application (mAFA) screening App across China. After excluding 647,087 subjects without compatible devices, 2,852,374 subjects had rhythm monitoring data; of these, 157 subjects with unknown rhythm were excluded, and 2,852,217 subjects were entered into the final analysis. Among them, 961,931 subjects screened for both atrial fibrillation (AF) and obstructive sleep apnea (OSA) risk using photoplethysmography-based smart devices. Among these, 6,120 subjects (0.6%, 89.2% male, mean age 53 ± 15 years) were monitored with suspected AF

In brief, the subjects aged over 18 years freely downloaded the AF screening app from Huawei App stores across China. After being acquainted with the study design, giving electronic informed consent, and having matched compatible smart devices, the subjects were entered into the study. Subjects aged <18 years or with the inability to use smartphones or devices were excluded. Adult subjects who signed electronic informed consent and matched compatible smart devices entered into this study between October 26, 2018, and December 1, 2021 (Figure 1). The study protocol is available in the Supplemental Appendix.

This study was approved by the Central Medical Ethic Committee of Chinese PLA General Hospital (approval number: S2017-105-02) and registered at the ChiCTR (Chinese Clinical Trial Registry) website (ChiCTR-OOC-17014138). Electronic written informed consent was obtained from all participants at enrollment.

### AF screening, confirmation, and follow-up

The algorithm with a PPG green light signal has been developed for screening suspected AF.^8^^,^^9^ At least 14-day monitoring using PPG-smart devices was proposed. Periodic measurements were to be automatically taken every 10 minutes, and 60-second PPG signals were to be continuously collected. Moreover, individuals could initiate active measurements at rest, and 45-second PPG signals were collected. The subjects who received a notification of suspected AF could freely decide whether to be followed up for confirmation of the diagnosis of AF by the mAFA Telecare team. Once they choose “Yes” on the AF screening App, with regard to the question of whether they would like to be followed up by mAFA Telecare team or not, the health providers in the mAFA Telecare center would contact him or her to confirm the diagnosis of AF to the network hospitals. AF was further diagnosed with clinical evaluation, 12-lead ECG, or 24-hour Holter monitoring by health providers from the mAFA Telecare team and network hospitals. The screened AF patients were considered for the mAFA II program to receive mHealth technology supported AF integrated care using the amfAR App, which was Avoid stroke with anticoagulants, Better symptom management, Cardiovascular and other comorbidities risk management, together with educational programs.^11^

### Definition of main cardiovascular risk factors

Study participants were required to fill out a questionnaire about palpitations and cardiovascular risk factors, using the AF screening App. Collected data on user-reported risk profiles are summarized as follows:

i. Hypertension was defined as a resting blood pressure ≥140 mm Hg systolic and/or ≥90 mm Hg diastolic on at least 2 occasions, or those having current antihypertensive drug treatment.
ii. Coronary artery disease (CAD) was defined as prior myocardial infarction, angina pectoris, percutaneous coronary intervention, or coronary artery bypass surgery.
iii. Heart failure was defined as the presence of signs and symptoms of either right ventricular failure (elevated central venous pressure, hepatomegaly, dependent edema) or left ventricular failure (exertional dyspnea, cough, fatigue, orthopnea, paroxysmal nocturnal dyspnea, cardiac enlargement, rales, gallop rhythm, pulmonary venous congestion) or both, diagnosed by doctors.
iv. Obstructive sleep apnea (OSA) is the apnea caused by upper airway obstruction during sleep, associated with frequent awakening and often with daytime sleepiness.
v. Hyperthyroidism was defined by excessive functional activity of the thyroid gland with abnormal thyroid hormone profile.
vi. Diabetes was defined as a fasting blood sugar level of 126 mg/dL (7 mmol/L) or higher, a random blood sugar level of more than 200 mg/dL (11.1 mmol/L).

When the study participants filled in the questionnaire, these risk factors were required to be confirmed by doctors.

### Sleep apnea screening with PPG smarter

A machine-learning model with PPG signals, including green light, infrared light, and red light sources, has been developed to monitor blood oxygen saturation.^12^ Compared with a home sleep apnea test, the PPG algorithm based on smart devices detected moderate-to-severe OSA patients (apnea hypopnea index [AHI] ≥15), with the accuracy, sensitivity, and specificity of 87.9%, 89.7%, and 86.0%, respectively.^12^ Subjects who had compatible Watch GT or higher (Huawei Technologies Co.) could freely decide whether they wanted to simultaneously receive sleep apnea screening plus AF screening using the AF screening App. The risk for sleep apnea monitored by PPG smart devices was grouped as normal, low risk, intermediate risk, and high risk. High risk of sleep apnea was defined as more than 80% monitoring measures with AHI ≥30 during sleep; intermediate-risk sleep apnea was defined as more than 80% monitoring measures with AHI ranged between 15-30 during sleep; and low-risk sleep apnea was defined as more than 80% monitoring measures with AHI ranged between 5-15 during sleep. All others were classified as normal.

### Statistical analysis

Continuous variables were tested for normality by the Kolmogorov-Smirnov test. Data with a normal distribution were presented as mean ± SD. Data with a non-normal distribution were presented as median (IQR).

The monitoring time, defined as time from first measurement to last measurement, were calculated. The proportion of detected AF was investigated in the first 14 monitoring days, third to fourth week, then monthly during the first year, followed by the second year, third year, and over 3 years.

Subjects were stratified according to the enrolled year as follows: October 26, 2018 to December 31, 2018, January 1, 2019 to December 31, 2019, January 1, 2020 to December 31, 2020, and January 1, 2021 to December 1, 2021. Given there were only approximately 2 months of enrollment in 2018, the subjects enrolled in 2018 were merged into those enrolled in 2019 and were analyzed compared with those in 2020 and 2021, respectively. We analyzed trends in the proportion of suspected AF as monitored by PPG devices by age strata and year.

All the statistically significant variables at univariate analysis were included in the multivariable model to distinguish the independent predictors of detected prevalent AF episodes. A Cox proportional hazards model was used to analyze the association of enrolled year and detected AF episodes, after adjustment (for age, gender, area, palpitation symptoms, hypertension, diabetes, sleep apnea, CAD, hyperthyroidism, and heart failure) and adjusted HRs (95% CI) are presented.

A logistic multivariate regression analysis was used to assess the effects of the risk strata of sleep apnea on the detected prevalent AF episodes, among subjects who simultaneously received sleep apnea screening and AF screening using the AF screening App. The 95% CIs were calculated with the Wilson score method without continuity correction. A 2-sided *P* value of <0.05 was considered statistically significant. Statistical analyses were performed using IBM SPSS Statistics (version 25.0) and MedCalc 12.6.1.0 (MedCalc Software).

## Results

Between October 26, 2018, and December 1, 2021, there were 3,499,461 subjects (79.2% male, mean age 37 ± 15 years) who downloaded the mAFA screening App across China (Supplemental Figure 1). After excluding 647,087 subjects without compatible devices, 2,852,374 subjects had rhythm monitoring data; of these, 157 subjects with unknown rhythm were excluded, so 2,852,217 subjects (81.7% male, mean age 38 ± 13 years) were entered into the final analysis (Figure 1). OSA was the most common self-reported risk factor, followed by hypertension and diabetes mellitus (Table 1). Baseline characteristics of the 2,852,217 subjects are shown in Table 1.

**Table 1:** Baseline Characteristics of 2,852,217 Subjects With Smart Devices Between 2018 and 2021

	2018 (n = 25,782)^a^	2019 (n = 751,341)	2020 (n = 1,040,043)	2021 (n = 1,035,051)
Age, y	37 ± 17	36 ± 22	38 ± 13	38 ± 12
Male	23,407 (90.8)	624,974 (83.2)	847,394 (81.5)	833,062 (80.5)
User-reported risk profiles (N = 1,314,964)	11,738	331,909	522,171	449,146
Palpitation	3,298 (28.1)	101,482 (30.6)	156,839 (30.0)	134,979 (30.1)
OSA	3,763 (32.1)	111,064 (33.5)	172,010 (32.9)	144,982 (32.3)
Hypertension	1,930 (16.4)	52,771 (15.9)	87,022 (16.7)	79,160 (17.6)
Diabetes	439 (3.7)	12,620 (3.8)	21,873 (4.2)	20,714 (4.6)
CAD	362 (3.1)	9,767(2.9)	16,895 (3.2)	16,059 (3.6)
Heart failure	161 (1.4)	5,053 (1.5)	8,336 (1.6)	7,577 (1.7)
Hyperthyroidism	161 (1.4)	4,725 (1.4)	7,738 (1.5)	6,960 (1.5)

Values are mean ± SD, n (%), or n.

CAD = coronary artery disease; OSA = obstructive sleep apnea syndrome.

a In 2018, the study period ran from October 26, 2018 to December 31, 2018.

### Detected AF and monitoring time

There were 12,244 subjects (0.43%; 83.2% male, mean age 57 ± 15 years) who received a notification of suspected AF. Of these, the median monitoring time (defined as time from first measurement to last measurement) was 271 (IQR: 92 to 520) days, and the time from first monitored suspect AF episode to last measurement was 187 (IQR: 45 to 381) days.

**Figure 2:**
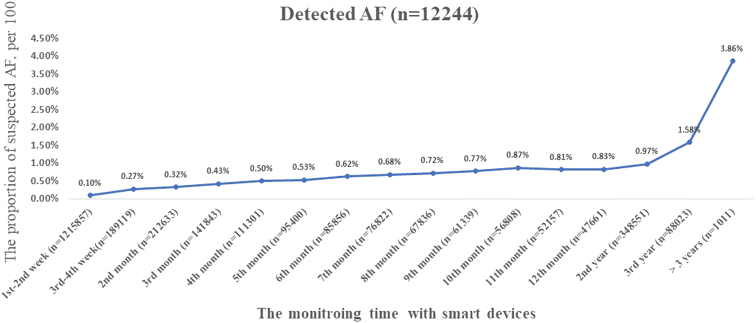
Proportion of Detected AF, in Relation to the Monitoring Time Monitoring time was defined as the time from first measurement to the last measurement. The proportion of detected atrial fibrillation (AF) was investigated in the first 14 monitoring days, third to fourth week, then monthly during the first year, followed by the second year, the third year, and over 3 years.

Detected AF episodes increased from 0.10% (1,219/1,215,857) in the first 14 days, to 0.83% (395/47,661) in the 12th month, 0.97% (3,392/348,551) in the second year, 1.58% (1,391/88,023) in the third year, and to 3.86% (39/1,011) at over 3 years (*P* for trend <0.001) (Figure 2).

### Detected AF stratified by age strata

The proportion of detected AF increased with ageing, from 0.10% aged 18 to 39 years, 0.42% aged 40 to 54 years, 1.79% aged 55 to 64 years, 3.79% aged 65-74 years, 6.96% ages 75 to 84 years, and 7.26% ages over 85 years (*P* for trend <0.001). The proportion of suspected AF in relation to age strata and year is summarized in Supplemental Table 1.

### Univariable and multivariable analyses

Male sex, age strata, heart failure, hypertension, hyperthyroidism, diabetes, and OSA, contributed to the risk for detected AF, with HRs between 1.02 and 2.75 (all *P* < 0.001) (Table 2).

**Table 2:** Univariable and Multivariable HRs for Detected AF in 2,852,217 Subjects

	Univariable		Multivariable	
	HR (95% CI)	*P* Value	HR (95% CI)	*P* Value
Male	1.13 (1.07-1.19)	<0.001	1.51 (1.44-1.59)	<0.001
Age	2.77 (2.74-2.81）	<0.001	2.72 (2.68-2.75)	<0.001
Area	1.08 (1.07-1.10)	<0.001	1.06 (1.05-1.07)	<0.001
Palpitation	2.47 (2.38-2.60）	<0.001	2.14 (2.05-2.24)	<0.001
Heart failure	9.03 (8.43-9.66)	<0.001	2.51 (2.32-2.72)	<0.001
CAD	6.78 (6.42-7.16)	<0.001	1.04 (0.96-1.11)	0.29
Hypertension	2.63 (2.52-2.74)	<0.001	1.08 (1.03-1.14)	0.001
Hyperthyroidism	2.37 (2.08-2.69)	<0.001	1.19 (1.04-1.35)	0.01
Diabetes	3.01 (2.80-3.22)	<0.001	1.09 (1.01-1.17)	0.02
OSA	1.26 (1.21-1.32)	<0.001	1.14 (1.09-1.20)	<0.001
Year				
2018-2019	Reference		Reference	
2020	1.56 (1.48-1.63)	<0.001	1.34 (1.27-1.40)	<0.001
2021	1.94 (1.85-2.05)	<0.001	1.67 (1.59-1.76)	<0.001

Detected AF in 12,244 patients. Age (per year) was as a continuous variable into the multivariable hazard model.

Abbreviations as in Table 1.

**Figure 3:**
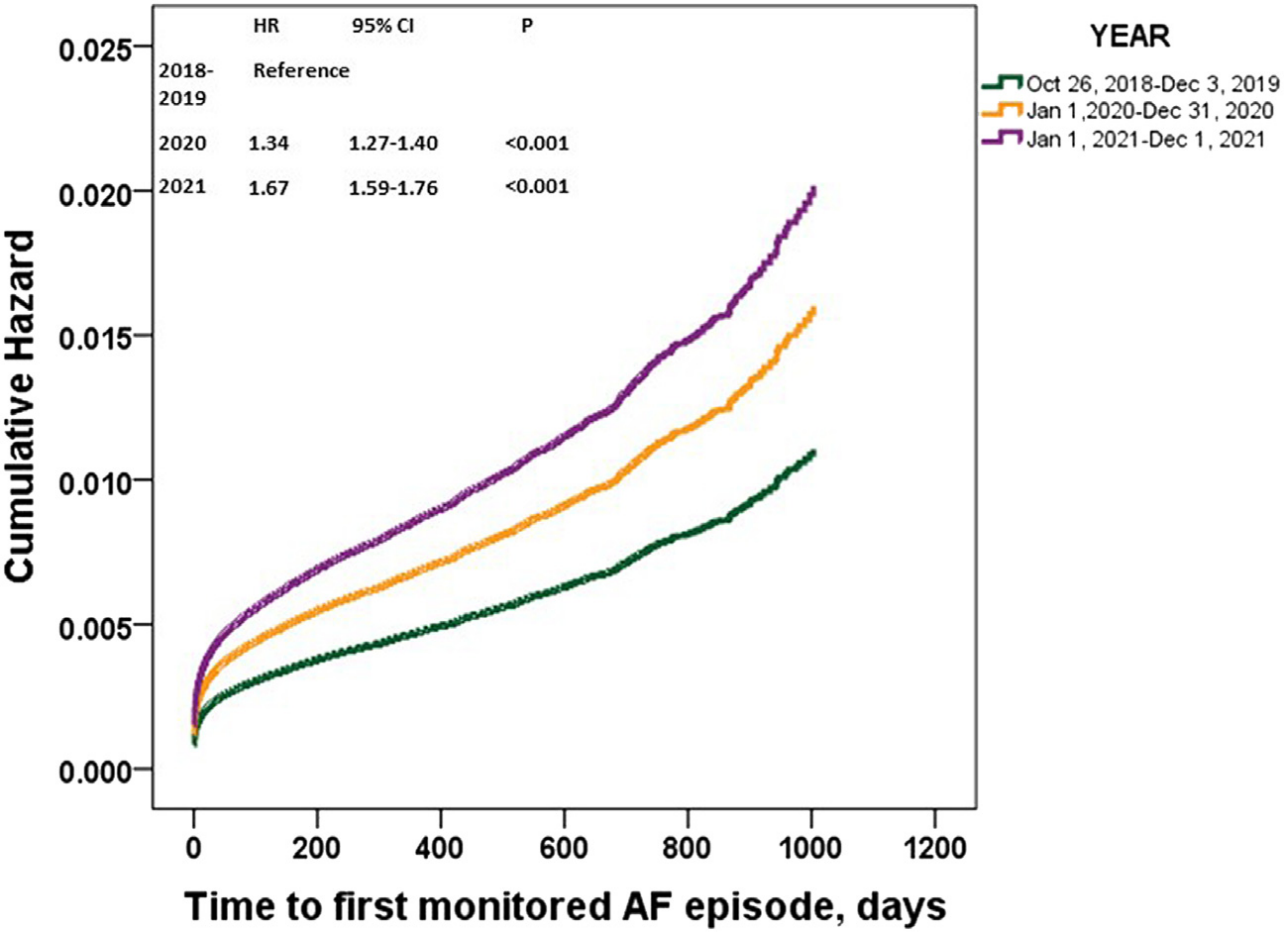
Cumulative Risk of Monitored AF Trends in age, gender-adjusted HRs of prevalent monitored atrial fibrillation (AF) were investigated across time periods using a Cox proportional hazards model. In this model, baseline confounders, including the area (Northeast, North China, East China, South China, Central China, Northwest, and Southwest), palpitation, hypertension, diabetes, obstructive sleep apnea syndrome, coronary artery disease, hyperthyroidism, and heart failure were adjusted.

The increased age- and gender-adjusted trend in period prevalence of first monitored AF by PPG smart device was significant (all *P* < 0.001). When compared with 2018, the risk for monitored prevalent AF increased significantly for subjects when monitoring started in 2020 (adjusted HR: 1.34; 95% CI: 1.27-1.40; *P* = 0.01) or in 2021 (adjusted HR: 1.67; 95% CI: 1.59-1.76; *P* < 0.001), after adjustment for age, gender, area, user-reported risk profiles (palpitation symptoms, hypertension, diabetes, OSA, CAD, hyperthyroidism, and heart failure) (Table 3, Figure 3).

### OSA risk and detected AF

There were 961,931 subjects (86.9% male, mean age 37 ± 14 years) who were screened for both AF and OSA risk using PPG smart devices. Among these, 6,120 subjects (0.6%, 89.2% male, mean age 53 ± 15 years) were monitored with suspected AF.

Of the 961,931 subjects, 18,032 (1.9%, 97.8% male, mean age 44 ± 17 years) were identified as high risk for OSA, and 311 (1.7%, 95.5% male, mean age 53 ± 13 years) were identified with suspected AF. The distribution of the risk of OSA is shown in Supplemental Figure 2.

**Figure 4:**
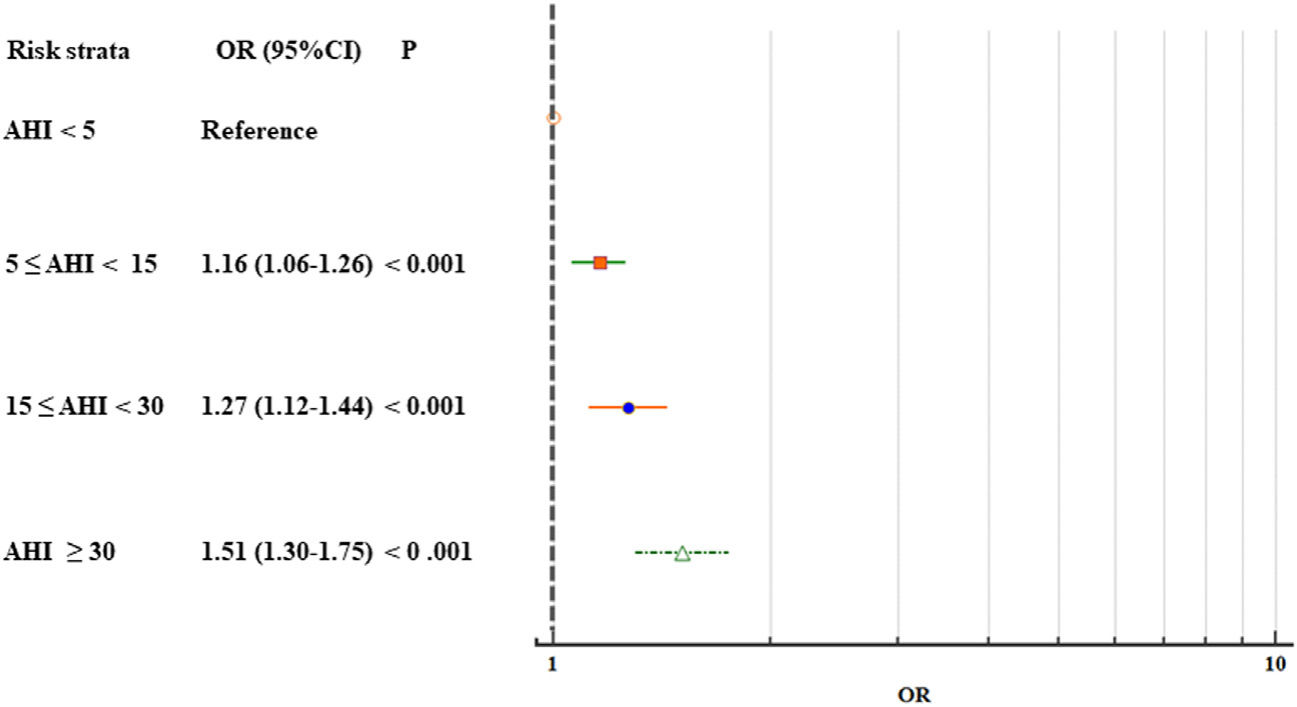
ORs of Detected Atrial Fibrillation by the Risk Strata of OSA Of the 961,931 subjects who screened for both atrial fibrillation and obstructive sleep apnea, 18,032 (1.9%, 97.8% male, mean age 44 ±17 years) were identified as high risk for obstructive sleep apnea (>80% monitoring measures with apnea hypopnea index [AHI] ≥30 during sleep), which resulted in a 1.5-fold increase (95% CI: 1.30- to 1.75-fold) in prevalent atrial fibrillation.

On multivariate analysis, high-risk sleep apnea (>80% monitoring measures with AHI ≥30 during sleep) increased the risk of AF detection by 1.51-fold (95% CI: 1.30- to 1.75-fold), after adjustment of age, sex, body mass index, hypertension, diabetes mellitus, CAD, heart failure, and hyperthyroidism (Figure 4).

### Confirmation of AF diagnosis

There were 2,447 subjects who refused to be followed up when they received the notification of suspected AF. Among 5,227 subjects (53.3%, 5,227/9,797) subjects effectively followed-up, 4,903 subjects (93.8%, 4,903/5,227) were confirmed with the diagnosis of AF by clinical evaluation and 12-lead ECG or 24-hour Holter, by the mAFA Telecare Team and doctors.

## Discussion

In this large, prospective, population-based, consumer-led screening study using smartwear conducted over 3 years, our main findings are as follows: 1) PPG-based smart devices can facilitate screening for AF with >93% confirmation of suspected AF episodes; 2) a consumer-led screening approach demonstrates the increased risk for detecting prevalent AF episodes over time; 3) male sex, age, user-reported risk factors (heart failure, hypertension, hyperthyroidism, diabetes mellitus, OSA) independently predicted detected AF episodes; and 4) OSA (as detected by smartwear) was the most common reported risk factor, whereas high-risk OSA (more than 80% monitoring measures with AHI ≥30 during sleep) resulted in a 1.5-fold increase in prevalent AF.

### Consumer-led AF screening approach in a low-risk population

Our prior validation studies reported that the diagnostic sensitivity, specificity, positive predictive value, negative predictive value, and accuracy of mobile phones with PPG for AF detection were over 94%, compared with 12-lead ECG.^8^^,^^9^ In this present study involving 3 million subjects with a mean age of 37 years over 3 years, the confirmation of suspected AF by PPG devices remained over 93%, consistent with the previous report from the Mafi-II trial Long-term Extension Cohort.^13^ In the WATCH AF trial (SmartWATCHes for Detection of Atrial Fibrillation) using a smartwatch-based PPG algorithm, there was a sensitivity of 93.7%, a specificity of 98.2%, a positive predictive value of 97.8%, and a negative predictive value of 94.7%, respectively, as well as an overall accuracy of 96.1% for AF detection among subjects, with a mean age 76 years.^14^ Thus, affordable, easy-for-use, consumer-led PPG-based smartwear can be a good screening tool, not only for an elderly population with comorbidities, but also for mass screening in the low-risk general population, with more prolonged, frequent monitoring.

### Risk Factors for Developing AF

We observed an increased adjusted risk for detecting prevalent AF episodes over time, and male sex, age, heart failure, hypertension, hyperthyroidism, diabetes mellitus, and OSA contributed to the risk for detected AF, with HRs between 1.04 and 2.72. Indeed, population-based studies have identified numerous factors that increase AF susceptibility. The available studies have established that advanced age, male sex, obesity, diabetes mellitus, OSA, elevated blood pressure, and heart failure all predispose to AF, with HRs ranged from 1.13 to 2.18.^15^

The increased secular trends on detected prevalent AF are perhaps affected by increasing cardiovascular risk factors that enhance AF susceptibility in the Chinese population. Over the last decade, obesity has increased by 67.6% in the Chinese adult population, whereas the mean physical activity has reduced from 385.9 MET h/7 d to 212.8 MET h/7 d.^16^ Growing trends are also seen in the prevalence of hypertension, which has increased to 27.9% in adults over 18 years,^16^ whereas the all-age prevalence of diabetes rose from 3.7% to 6.6% from 1990 to 2016.^17^ On the other hand, higher levels of cardiovascular health are associated with decreased risk of developing AF.^18^

Moreover, OSA was the most popular user-reported risk factor in the present screening study. A recent meta-analysis of the global prevalence and burden of OSA demonstrated that the prevalence was highest in China, followed by the United States, Brazil, and India, with an estimated 936 million (95% CI: 903-970 million) men and women aged 30 to 69 years having mild-to-severe OSA (AHI ≥5), and 425 million (95% CI: 399-450 million) having moderate-to-severe OSA (AHI ≥15) globally.^19^ The present large, population-based screening study found a 1.5-fold increase in the prevalent AF with high-risk OSA (detected via smartwear), suggesting the need to control risk factors that increase AF susceptibility.

### Implications for clinical practice

Paroxysmal AF is missed in clinical practice because many patients with the condition are asymptomatic, and AF episodes frequently occur outside the monitoring windows captured by 12-lead ECGs. Indeed, prolonged monitoring is likely to improve the detection of paroxysmal AF.^20^ The first detection time of AF burden of <50% per 24 hours was 4 days by active measurement and 2 days by periodic measurement.^9^ Instead of one-off ECGs, PPG-based smart devices allow continuous monitoring and would permit a much earlier detection of paroxysmal AF or asymptomatic AF, allowing the timely introduction of therapies to protect patients, not only from the consequences of the arrhythmia, but also from progression of AF from an easily treated condition to an utterly refractory problem.

Indeed, the lifestyle and risk factor modification interventions are increasingly associated with reduced AF burden.^21^ However, these lifestyle factors cannot be considered in isolation; for example, OSA would contribute to hypertension, diabetes, and HF. Hence, an integrated care approach would be required to fully implement clustered risk management in the AF patient, not just focusing on individual risk factors. The use of smart technology may support implementation of the integrated approach aimed at both primary and secondary prevention.^22^ Also, the possibility of smartwear to detect risk factors such as OSA and patient-centered risk factor mitigation would be consistent with the move toward a more holistic or integrated care approach to AF management^23^ that is now recommended in guidelines.^24^ Adherence to such an integrated care approach has been associated with improved clinical outcomes.^25^

### Study Limitations

There were only 53.3% subjects with identified suspected AF who were effectively followed up by mAFA Telecare Team and doctors. One reason was that this was a large, prospective, consumer-led screening study involving 3.5 million subjects over 3 years. Also, this was a relatively low-risk population, with a mean age of 38 years. Though suspected AF was monitored, they were not willing to have further confirmation, possibly because of their asymptomatic status. To avoid the underlying selection bias, we analyzed trends in the proportion of suspected AF as monitored by PPG devices by age strata and year. Besides, given this was a large, prospective, consumer-led screening study, we cannot confirm that the first detected AF episode was a new AF episode, or paroxysmal episode, or asymptomatic AF. Moreover, the COVID-19 pandemic greatly changes the lifestyle. It is unclear what the underlying impact of COVID-19 might bring up for the prevalence change of monitored AF and deserves to be further investigated.

## Conclusions

PPG-based smart devices can facilitate screening for AF with >93% confirmation of suspected AF episodes, even for the low-risk general population, with more prolonged monitoring. This consumer-led AF screening approach highlights the increased risk for detecting prevalent AF episodes over time and the need for modification of OSA and other risk factors that increase AF susceptibility (Central Illustration).

Perspectives **COMPETENCY IN PATIENT CARE:** The photoplethysmography-based smart devices, which can facilitate screening for AF with >93% confirmation of detected AF episodes even for the low-risk general population, demonstrated the increased risk for detecting prevalent AF episodes over time; detected high-risk OSA resulted in a 1.5-fold increase in prevalent AF, highlighting the need for modification of OSA and other risk factors that increase AF susceptibility.

**TRANSLATIONAL OUTLOOK:** Future studies will be needed to move toward a “smart” technology supported implementation of clustered risk management in the AF patient.

**Central Illustration:**
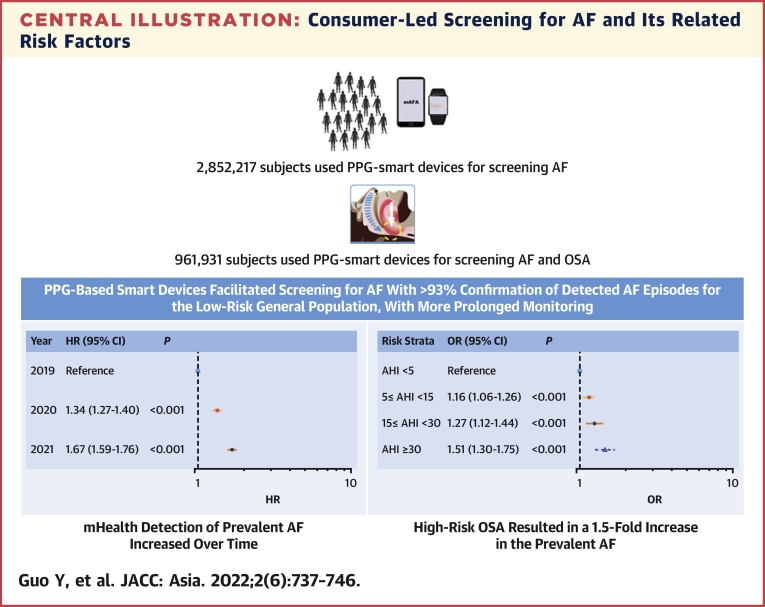
Consumer-Led Screening for AF and Its Related Risk Factors In the present largest screening study involving 2,852,217 subjects over 3 years for atrial fibrillation (AF) and 979,013 subjects for both AF and obstructive sleep apnea (OSA), photoplethysmography (PPG)-based smart devices can facilitate screening for AF with >93% confirmation of detected AF episodes, even for the low-risk general population, with more prolonged monitoring. High risk for OSA resulted in a 1.5-fold increase in the prevalent AF. This consumer-led AF screening approach highlights the increased risk for detecting prevalent AF episodes over time and the need for modification of OSA and other risk factors that increase AF susceptibility. AHI = apnea hypopnea index; mAFA = mobile Atrial Fibrillation Application.

## Funding Support and Author Disclosures

This research project was funded by the National Natural Science Foundation of China (82170309). This study was an investigator-initiated project, with limited funding by independent research and educational grants. Dr Lip has been a consultant and speaker for BMS/Pfizer, Boehringer Ingelheim, and Daiichi Sankyo, for which he has received no personal fees. All other authors have reported that they have no relationships relevant to the contents of this paper to disclose.
