# Supplementary material for: Consumer-Led Screening for Atrial Fibrillation: A Report From the mAFA-II Trial Long-Term Extension Cohort
Source: JACC Asia. 2022 Nov 1;2(6):737–46. doi: 10.1016/j.jacasi.2022.07.006 (PMC9700030; doi:10.1016/j.jacasi.2022.07.006)
Supplement: Supplemental Data [file mmc1.docx]

**Supplemental Table 1. The Proportion of Monitored AF in Relation to Age Strata and Year**

| **Year** | **2018-2019** | | | | **2020** | | | | **2021** | | | |
| --- | --- | --- | --- | --- | --- | --- | --- | --- | --- | --- | --- | --- |
| Age strata | Monitored AF, (n) | Number, (n) | Rate, (%) | 95% CI | Monitored AF, (n) | Number, (n) | Rate, (%) | 95% CI | Monitored AF, (n) | Number, (n) | Rate,  (%) | 95% CI |
| Age 18-39 | 519 | 504 708 | 0.10% | 0.09%-0.11% | 749 | 632 374 | 0.12% | 0.11%-0.13% | 502 | 630 920 | 0.08% | 0.07%-0.09% |
| Age 40-54 | 957 | 216 907 | 0.44% | 0.41%-0.47% | 1293 | 310 113 | 0.42% | 0.39%-0.44% | 1190 | 298 999 | 0.40% | 0.38%-0.42% |
| Age 55-64 | 703 | 36 296 | 1.94% | 1.80%-2.08% | 1176 | 63 010 | 1.87% | 1.76%-1.98% | 1161 | 70 625 | 1.64% | 1.55%-1.74% |
| Age 65-74 | 589 | 15 018 | 3.92% | 3.62%-4.24% | 1046 | 26 600 | 3.93% | 3.71%-4.17% | 948 | 26 497 | 3.58% | 3.36%-3.81% |
| Age 75-84 | 234 | 3569 | 6.56% | 5.79%-7.42% | 491 | 6638 | 7.40% | 6.79%-8.05% | 458 | 6802 | 6.73% | 6.16%-7.35% |
| Age >85 | 41 | 625 | 6.56% | 4.87%-8.78% | 107 | 1308 | 8.18% | 6.81%-9.79% | 80 | 1208 | 6.62% | 5.35%-8.17% |
| Total | 3043 | 777 123 | 0.39% | 0.38%-0.41% | 4862 | 1 040 043 | 0.47% | 0.45%-0.48% | 4339 | 1 035 051 | 0.42% | 0.41%-0.43% |
| *P* |  |  | < .001 |  |  |  | < .001 |  |  |  | < .001 |  |

* CI: confidential intervals.

**Supplemental Figure 1: Enrolment Into the HUAWEI HEART STUDY**

* Red arrow means the end study date of reported Huawei Heart Study (Guo Y，et al. JACC. 2019).

**Supplemental Figure 2.** **The Distribution of the Risk of OSAS Monitored by PPG Algorithm (n=962,087)**

***** OSAS**:** obstructive sleep apnea syndrome. AHI: apnea hypopnea index.

**Mafi II investigators**

**Executive Steering Committee:**

| Yutao Guo | Chinese PLA General Hospital, Beijing, China (Co-Chair) |
| --- | --- |
| Gregory Y H Lip | Institute of Cardiovascular Sciences, University of Birmingham, UK; Liverpool Centre for Cardiovascular Science, University of Liverpool, UK (Co-chair) |
| Deirdre A. Lane | Liverpool Centre for Cardiovascular Science, University of Liverpool, UK |
| Yundai Chen | Chinese PLA General Hospital, Beijing, China |
| Liming Wang | The National Center for Chronic and Noncommunicable Disease Control and Prevention, Chinese Center for Disease Control and Prevention, Beijing, China |

**Steering committee:**

| Jens Eckstein | University Hospital Basel, Switzerland |
| --- | --- |
| G Neil Thomas | Institute of Applied Health Research, University of Birmingham, United Kingdom |
| Liu Tong | The Second Hospital of Tianjin Medical University, Tianjin, China |
| Feng Mei | Shanxi Dayi Hospital, Taiyuan, Shanxi, China |
| Liu Xuejun | Affiliated First Hospital, Shanxi Medical University, China |
| Li Xiaoming | Cardiovascular Disease Hospital of Shanxi Province, China |
| Shan Zhaoliang | PLA General Hospital, Beijing, China |
| Shi Xiangming | PLA General Hospital, Beijing, China |
| Zhang Wei | PLA Army General Hospital, Beijing, China |
| Xing Yunmi | Beijing Friendship Hospital, Capital Medical University, Beijing, China |
| Wen Jing | Beijing Haitians Hospital, Beijing, China |
| Wu Fan | Tianjin Medical University General Hospital, Tianjing, China |
| Yang Sitong | The First Affiliated Hospital, Ji Lin University, Ji Lin, China |
| Jin Xiaoqing | Tongji Hospital, Tongji Medical College, Huazhong University Of Science & Technology, Wuhan, China |
| Yang Bo | Xiangya Hospital Central South University, Changsha, China |
| Bai Xiaojuan | ShengJing Hospital of China Medical University, Shengyang, China |
| Jiang Yuting | Suqian Hospital, Jiangsu, China |
| Liu Yangxia | General Hospital of Shengyang Military, Shengyang, China |
| Song Yingying | Bozhou Renmin Hospital, Anhui, China |
| Tan Zhongju | The First Hospital of Zhejiang Province, Hangzhou, China |
| Yang Li | Yunnan Cardiovascular Hospital, Kunming, China |
| Luan Tianzhu | The First Affiliated Hospital of Haerbing Medical University, Haerbing, China |
| Niu Chunfeng | The Second Affiliated Hospital of Haerbing Medical University, Hearing, China |
| Zhang Lili | The Fourth Affiliated Hospital of herbing Medical University, Haerbing, China |
| Li Shuyan | The First Affiliated Hospital, Ji Lin University, Ji Lin, China |
| Wang Zulu | General Hospital of Shengyang Military, Shengyang, China |
| Xv Bing | The First People's Hospital of Shengyang, Shengyang, China |
| Liu Liming | The Second Affiliated Hospital of Shengyang Medical University, Shengyang, China |
| Jin Yuanzhe | The Fourth Affiliated Hospital of China Medical University, Shengyang, China |
| Xia Yunlong | The First Affiliated Hospital of Dalian Medical University, Dalian, China |
| Chen Xiaohong | The People's Hospital of Liaoning Province, Shengyang, China |
| Wu Fang | Rui Jin Hospital, Tong university School of Medicine, Shanghai, China |
| Zhong Lina | The Affiliated Hospital of Qingdao University, Qingdao, China |
| Sun Yihong | China-Japan Friendship Hospital, Beijing, China |
| Jia shujie | Beijing Anzhen Hospital, Capital Medical University, Beijing, China |
| Li Jing | Xuanwu Hospital Capital Medical University, Beijing, China |
| Li Nan | The Third People’s Hospital of Dalian, Dalian, China |
| Li shijun | Dalian Municipal Central Hospital Affiliated of Dalian Medical University, Dalian, China |
| Liu huixia | Guangdong Academy of Medical Sciences Guangdong General Hospital, Guangdong, China |
| Li Rong | The First Affiliated Hospital of Guangzhou University of Traditional Chinese Medicine, Guangzhou, China |
| Liu Fan | The Second Hospital of Hebei Medical University, Hebei, China |
| Ge qingfeng | North China University Science And Technology Affiliated Hospital |
| Guan tianyun | The Second Hospital of Jilin University, Jilin, China |
| Wen Yuan | The Second Affiliated Hospital of Nanchang University, Nanchang, China |
| Li Xin | BenQ Hospital affiliated to Nanjing Medical University, Nanjing, China |
| Ren Yan | Ruijin Hospital, Shanghai Jiao Tong University School of Medicine |
| Chen Xiaoping | Taiyuan City Central Hospital, Taiyuan, China |
| Chen ronghua | Tangshan People's Hospital, Tangshan ,China |
| Shi Yun | Tianjin Fourth Central Hospital ,Tianjin, China |
| Zhao yulan | The Second Affiliated Hospital of Zhengzhou University, Zhengzhou, China |
| Shi haili | Zhengzhou Central Hospital Affiliated to Zhengzhou University, Zhengzhou, China |
| Zhao yujie | Zhengzhou Seventh People's Hospital, Zhengzhou, China |
| Wang quanchun | Shenyang Fifth People's Hospital, Shenyang, China |
| Sun weidong | Taian City Central Hospital, Taian, China |
| Wei Lin | Harbin First Hospital, Harbin, China |

**Data Safety Monitoring Board:**

| Esther Chan | The University of Hong Kong, Hong Kong, China |
| --- | --- |
| Shan Guangliang | Department of Epidemiology and Statistics, Institute of Basic Medical Sciences, Peking Union Medical College, Beijing, China |
| Yao Chen | Peking University Clinical Research Institute, Beijing, China |
| Zong Wei | China Foreign Affairs University, Beijing, China |
| Chen Dandi | West China School of Public Health, Chengdu, China |

**Clinical events committee:**

| Han Xiang | Department of Neurology, Huashan Hospital of Fudan University, Shanghai, China |
| --- | --- |
| Xu Anding | Department of Neurology, the First Affiliated Hospital of Jinan University, Guang Zhou, China |
| Fan Xiaohan | Fuwai Hospital, Chinese Academy of Medical Sciences, Beijing, China |
| Yu Ziqiang | Institute of Blood Research of Jiangsu Province, China |
| Gu Xiang | Department of Cardiology, People’s Hospital of Subei, Affiliated Hospital of YangZhou University, Jiangsu Province, China |
| Ge Fulin | Department of Gastroenterology, Chine PLA General Hospital, Beijing, China |

**AF Screening Protocol**

# Background

Atrial fibrillation (AF) is the most common arrhythmia with an increasing public health burden worldwide. The asymptomatic nature and paroxysmal frequency of AF lead to suboptimal early detection. A novel technology, photoplethysmography (PPG), has been developed for AF screening and AF-related risk factors, e.g. sleep apnea.

Our prior study validated the diagnostic ability of mobile phone and smart band apps with PPG compared to 12-lead electrocardiograms (ECG) in 112 consecutive inpatients. The diagnostic sensitivity, specificity, positive predictive value, negative predictive value, and accuracy of mobile phones with PPG for AF detection were over 94%. The feasibility of PPG-based smart devices for the detection of AF with continuing 14 -day monitoring was further investigated in 361 adult subjects in real-world settings. Smart devices demonstrated >91% predictive ability for AF. Upon the above evidence, PPG-based smart devices will be utilized for AF screening in general population of mAFA II program.

The mAFA II program is designed to investigate Mobile Health (mHealth) technology for improved screening and optimizing integrated care in atrial fibrillation, including Pre-mAFA phase of AF screening, also called HUAWEI HEART STUDY, using smart devices ((Huawei Technologies Co., Ltd., Shenzhen, China). This phase will investigate the incidence of AF identified with PPG-based screening strategy among general population, and then those with identified AF would be considered for entry into the mAFA II trial to validate the integrated ABC care-supported by mHealth technology in the management of AF.

# STUDY OBJECTIVES

## Primary Outcome

The primary endpoint of this study is the effectiveness of AF screening in a large population-based cohort using smart device–based PPG technology.

## Secondary Outcomes

Secondary endpoints comprise:

The prevalence of detected AF and AF-related risk factors

AF progression

Artificial intelligence machine-learning model for AF prediction

Characteristics and outcome of subjects with smart devices, vs. without smart devices, transferred into mAFA II trial

Cost

# STUDY design

In this cohort study of the first stage of the mAFA II program, subjects can monitor the pulse rhythm using AF screening App if they had smartphone and matched smart devices. Identified AF patients will be then transferred into a structured program of holistic and integrated care with mAFA App.

# STUDY POPULATION

## Inclusion criteria

The subjects aged over 18 years in China.

The subjects need to have compatible Huawei smart device(s) and a smartphone.

The use of the Huawei phone (Android 5.0 or higher), and one of following smart devices: Huawei Watch GT (version 1.0.3.52 or higher), Honor Watch (version 1.0.3.52 or higher), and Honor Band 4 (version 1.0.0.86 or higher).

## Excluded criteria

Age <18 years and inability to use smartphone or devices.

## AF detection

All subjects who are interested in the study will be informed of the study design and give their informed consent before entering the study. Adults downloading the AF screening app across the China will be enrolled into pre-MAFA study. At least 14-day monitoring with smart devices based on PPG (with the PPG algorithm developed by Huawei) is proposed.

The subjects could initiate rhythm monitoring with AF screening app using smart devices. The irregular pulse wave would be screened with active or periodic measuring using the PPG algorithm.

Subjects could initiate active measurements at rest, and 45-s PPG signals would be collected. Periodic measurements would be automatically taken every 10 min, and 60-s PPG signals would continuously be collected (Supplemental Figures 3 and 4. The discrimination rule of the PPG algorithm and notification of “suspected AF” is shown in Supplemental Figure 5. A notification of suspected AF would be delivered, once the proportion of “possible AF” episodes was 100%, when 10 measurements were initiated. In the case of >10 PPG measurements, the threshold T was set to ensure that the positive predictive value of making a decision was over 0.85, and the sensitivity would be as high as possible.

Threshold T could be adjusted to a more suitable value when enough study data were collected. The notification of suspected AF would also be delivered once the proportion of possible AF episodes over threshold T in the setting of PPG measurements was >10 (Supplemental Figure 5).


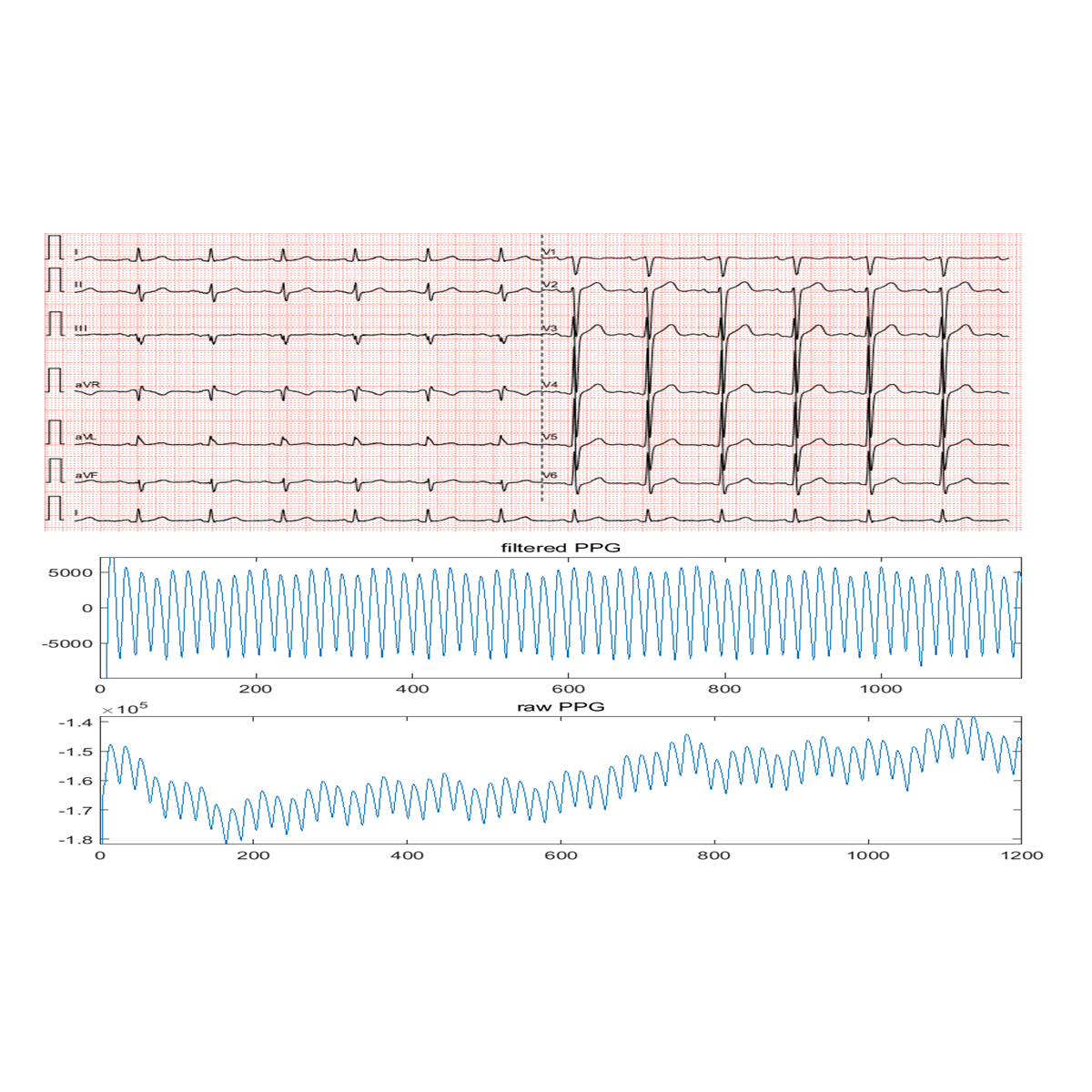


**Supplemental Figure 3: Electrocardiogram and Photoplethysmography in Patients With Sinus Rhythm**

**
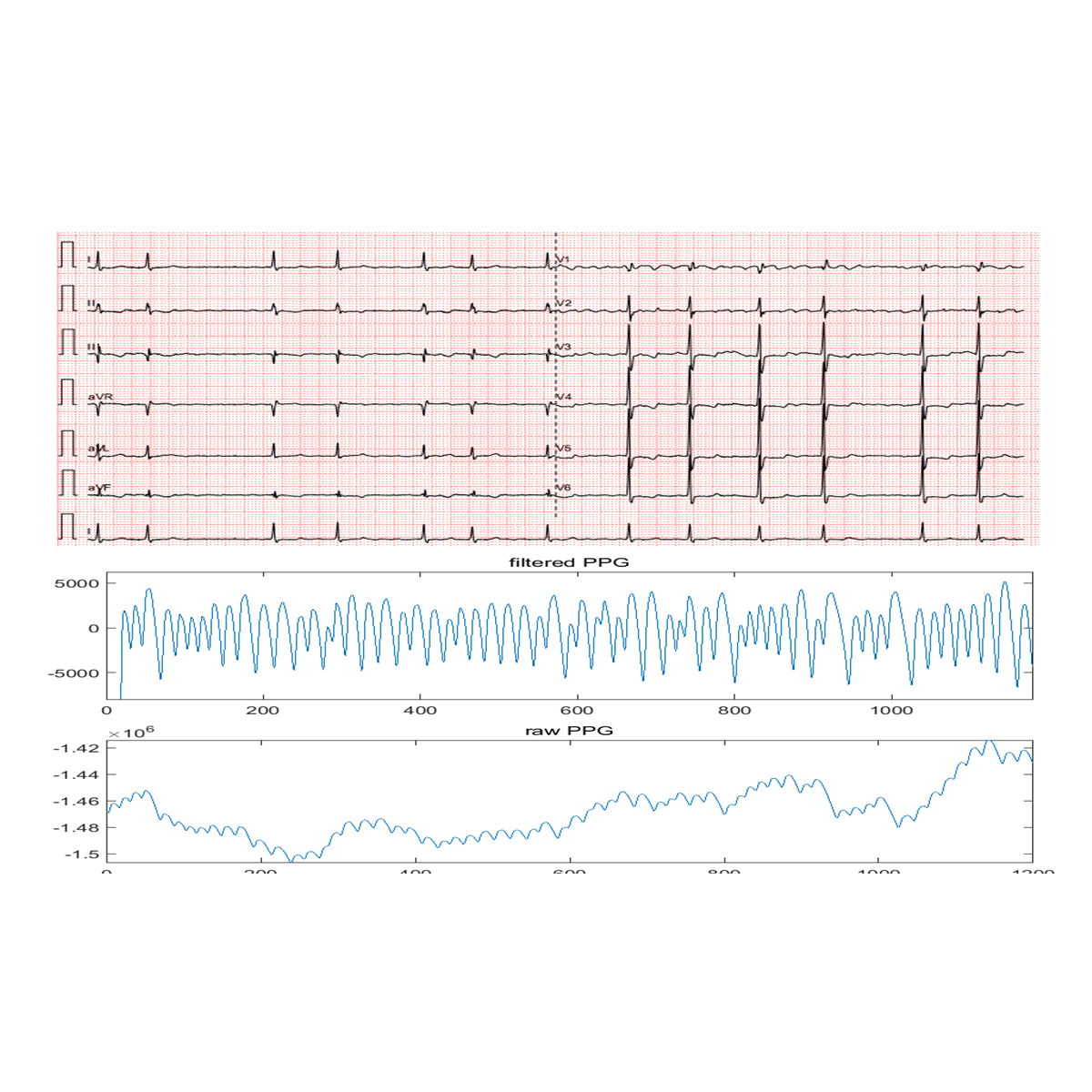
**

**Supplemental Figure 4: Electrocardiogram and Photoplethysmography in Patients With Atrial Fibrillation**


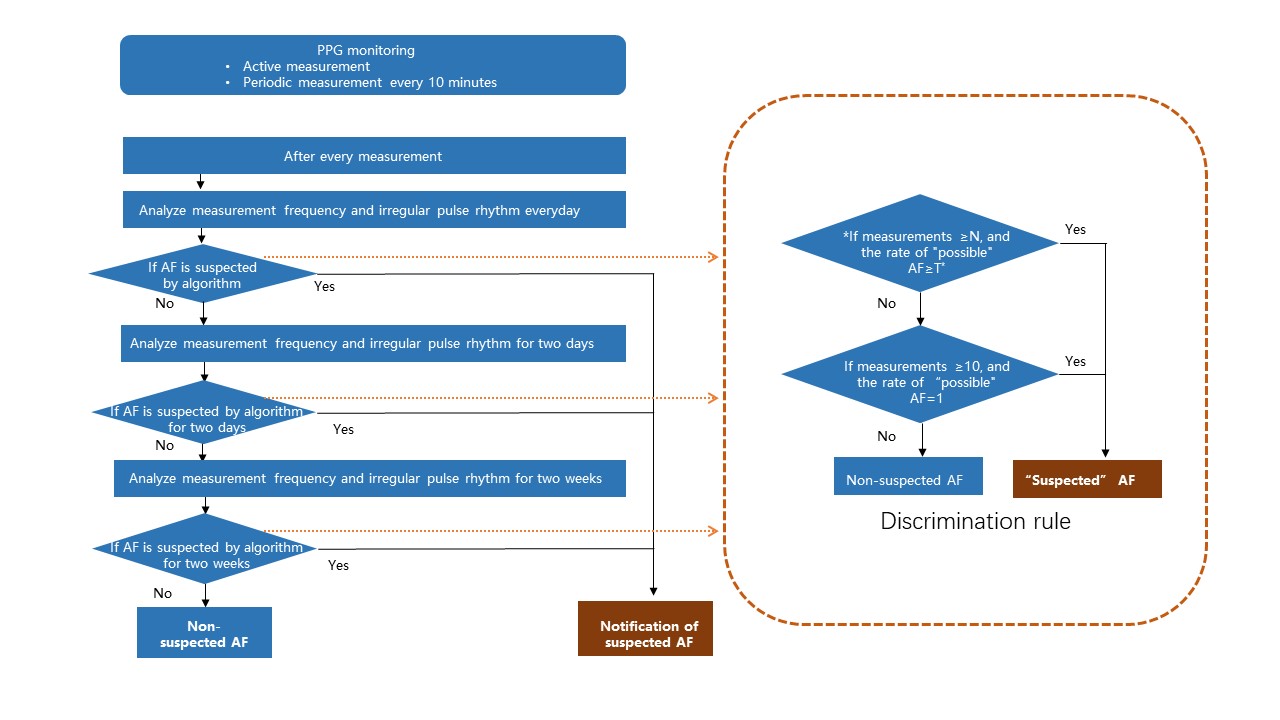


**Supplemental Figure 5： The Notification of Suspected Atrial Fibrillation (AF) by Algorithm. *N > 10, 0 < T < 1, see AF Detection in the Methods Section. PPG = photoplethysmography**

The subjects with suspected AF episodes using the PPG algorithm will be further confirmed by the health providers of the mAFA Telecare center and network hospitals, with clinical evaluation, ECG, or 24-h Holter monitoring (Supplemental Figure 6). Subjects with “identified AF” would be managed according to an app-based AF integrated care pathway approach, based on the ABC (Avoid Stroke, Better Symptom Management, and Cardiovascular Risk and Comorbidity Management) pathway.


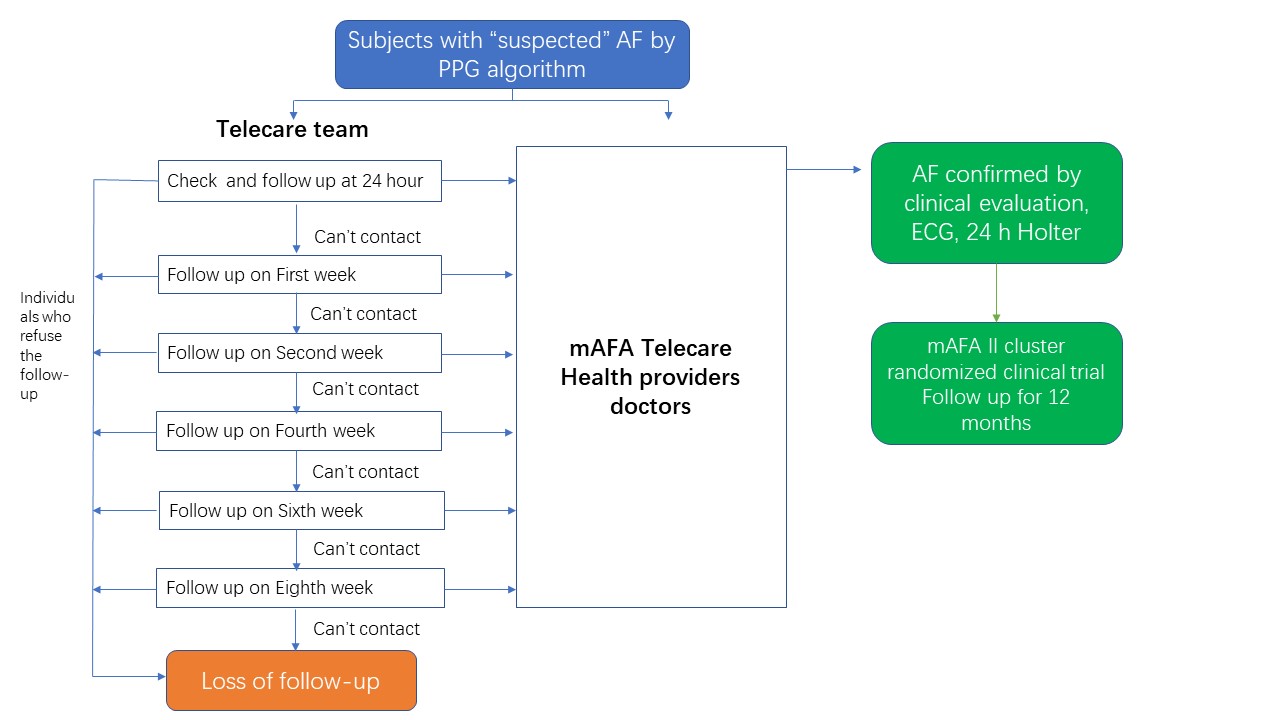


**Supplemental Figure 6： The Confirmation and Follow-Up of Individuals With “Suspected” AF**

## PPG-based smart devices screening obstructive sleep apnea

Subjects who had compatible Watch GT or higher (HUAWEI Device Co., Ltd.), could freely decide if they simultaneously received sleep apnea screening plus AF screening using AF screening App.

The OSA screening algorithm is embedded into the smartwatch. PPG signals are frequently recorded using a pulse oximeter to detect blood volume changes in the microvascular bed of the tissue. Compared to end-tidal carbon dioxide (EtCO2), the PPG-derived respiratory rate is more stable and less affected by the motion-induced artifact and patient talk. Thus, combined with pulse oximetry, PPG is a robust tool for screening early respiratory compromise. A smartwatch detects a user’s motions.

Using the spectra of PPG and acceleration signals, a rare while continuous activity in the patient was defined as “asleep,” and when a noticeable movement was detected, the patient was identified as “awake.” The data from sleep for >7 h were deemed reliable and will be used for screening the risk of sleep apnoea.

Using the machine learning algorithm and the overnight PPG signals, including green light, infrared light, and red-light sources, the initial screening model of sleep apnoea is established. The PRV characteristics were extracted from green light signals, blood oxygen saturation data were estimated and extracted from infrared and red-light signals, and the risk of sleep apnoea was evaluated by constructing a classification model based on the characteristics of pulse rate variability (PRV) and blood oxygen saturation. In addition, the acceleration signal from the wrist was used for an effective signal screening and an abnormal scene discrimination. Subsequently, respiratory waveforms were derived from PPG signals, sleep time was recorded, and apnoea hypopnea index (AHI) is calculated. Besides, 30% and 90% reduction in the respiratory waveforms for defining hypopnea and apnoea could be applied by the software, respectively

**
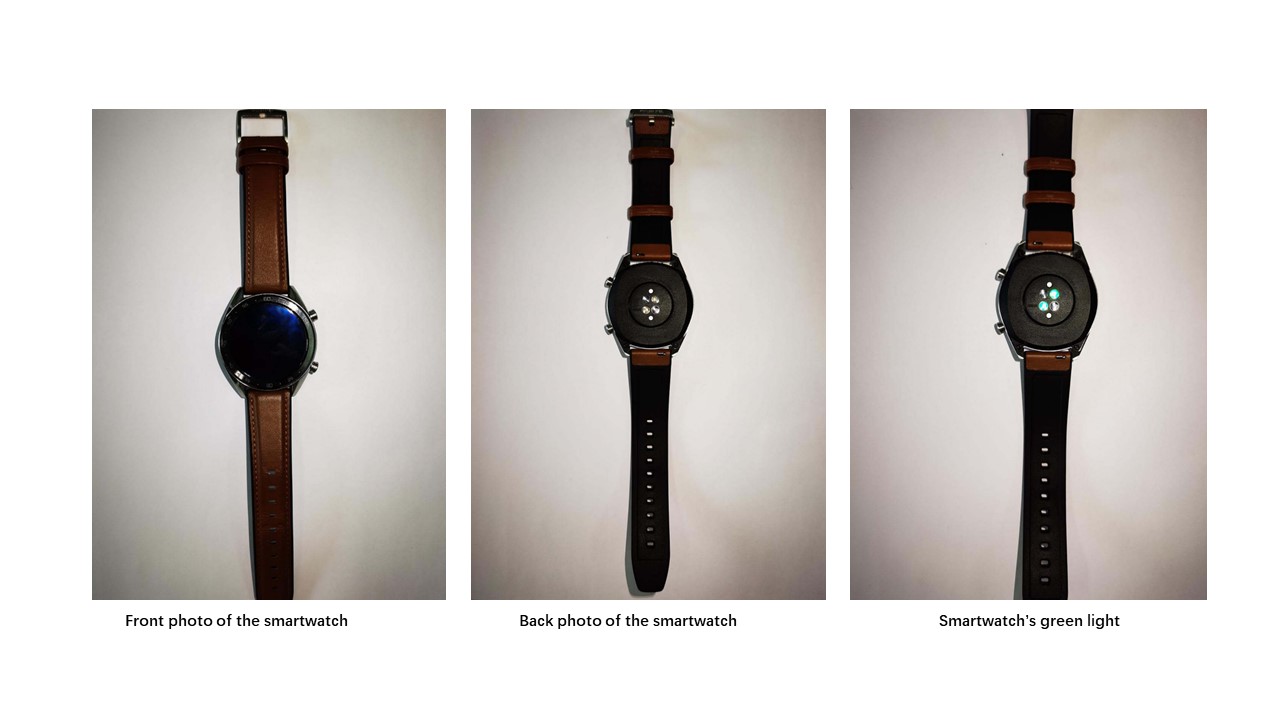
**

**Supplemental Figure 7： Smart device for screening sleep apnoea**

## Risk strata of detected obstructive sleep apnoea

The risk for sleep apnea monitored by PPG-smart devices was grouped as: normal, low-risk, intermediate risk, and high risk. ‘High-risk’ of sleep apnea was defined as more than 80% monitoring measures with AHI ≥ 30 during sleep; ‘Intermediate-risk’ sleep apnea was defined as more than 80% monitoring measures with 15 < AHI < 30 during sleep; and ‘Low-risk’ sleep apnea was defined as more than 80% monitoring measures with 5 < AHI ≤ 15 during sleep. All others were classified as the normal.

## Obstructive sleep apnoea diagnosis

The subjects with the risk for sleep apnoea using the PPG algorithm will be further confirmed by the health providers of the mAFA Telecare center and network hospitals, with the polysomnography (PSG) or home sleep apnoea test (HSAT).

# Statistical analysis

Continuous variables were tested for normality by the Kolmogorov-Smirnov test. Data with a normal distribution were presented as a mean (standard deviation, SD). Data with a non-normal distribution were presented as median (interquartile range, IQR) and were analyzed by using Kruskal-Wallis test. χ2 test was used for categorical variables.

The “irregular pulse rhythm” by PPG algorithm was observed, and the predictive ability of AF with PPG algorithm will be analyzed in comparison with the confirmed diagnosis of AF using clinical evaluation, ECG, or 24-h Holter by the health providers from the mAFA Telecare center and network hospitals. The proportion of detected AF and its-related risk factor, e.g. sleep apnoea, will be investigated.

A two-sided P-value < 0.05 was considered as statistically significant. The 95% confidential intervals (CIs) were calculated with Wilson score method without continuity correction. Statistical analysis was performed using IBM SPSS Statistics, version 25.0 (SPSS Inc.).
